# Construction of anoikis-related lncRNAs risk model: Predicts prognosis and immunotherapy response for gastric adenocarcinoma patients

**DOI:** 10.3389/fphar.2023.1124262

**Published:** 2023-02-28

**Authors:** Qinglin Li, Huangjie Zhang, Jinguo Hu, Lizhuo Zhang, Aiguang Zhao, He Feng

**Affiliations:** ^1^ Longhua Hospital, Shanghai University of Traditional Chinese Medicine, Shanghai, China; ^2^ Zhejiang Cancer Hospital, Hangzhou, Zhejiang, China; ^3^ Key Laboratory of Head and Neck Cancer, Translational Research of Zhejiang Province, Hangzhou, Zhejiang, China; ^4^ Hangzhou TCM Hospital Affiliated to Zhejiang Chinese Medical University, Hangzhou, Zhejiang, China; ^5^ Zhejiang Provincial People’s Hospital, Hangzhou, Zhejiang, China

**Keywords:** anoikis, lncRNAs, prognosis prediction, immunotherapy, gastric adenocarcinoma

## Abstract

**Background:** Anoikis acts as a programmed cell death that is activated during carcinogenesis to remove undetected cells isolated from ECM. Further anoikis based risk stratification is expected to provide a deeper understanding of stomach adenocarcinoma (STAD) carcinogenesis.

**Methods:** The information of STAD patients were acquired from TCGA dataset. Anoikis-related genes were obtained from the Molecular Signatures Database and Pearson correlation analysis was performed to identify the anoikis-related lncRNAs (ARLs). We performed machine learning algorithms, including Univariate Cox regression and Least Absolute Shrinkage and Selection Operator (Lasso) analyses on the ARLs to build the OS-score and OS-signature. Clinical subgroup analysis, tumor mutation burden (TMB) detection, drug susceptibility analysis, immune infiltration and pathway enrichment analysis were further performed to comprehensive explore the clinical significance.

**Results:** We established a STAD prognostic model based on five ARLs and its prognostic value was verified. Survival analysis showed that the overall survival of high-risk score patients was significantly shorter than that of low-risk score patients. The column diagrams show satisfactory discrimination and calibration. The calibration curve verifies the good agreement between the prediction of the line graph and the actual observation. TIDE analysis and drug sensitivity analysis showed significant differences between different risk groups.

**Conclusion:** The novel prognostic model based on anoikis-related lncRNAs we identified could be used for prognosis prediction and precise therapy in gastric adenocarcinoma.

## Introduction

Gastric cancer (GC) has been described as one of the most common cancers and the fourth leading cause of cancer-related death (Sung et al., 2021). There are more than one million new cases of GC all over the world each year, Chinese patients account for about 50 percent (Chen et al., 2016). In terms of pathological type, 90 percent of gastric cancer is stomach adenocarcinoma (STAD). Survival rate of patients with early-stage STAD has improved significantly over the past few decades, whereas those suffering from late-stage STAD with distant metastasis have a poor prognosis (Harada et al., 2017; Banks et al., 2019). As a consequence, it has great clinical significance to further explore the mechanism and stratify the risk of distant metastasis.

Metastasis of gastric cancer is a complicated process. Gastric cancer cells enter the abdominal cavity or circulatory/lymphatic system through migration and invasion to the basement membrane. In doing so, they have to be survival without touching the extra-cellular matrix (ECM) (Simpson et al., 2008). Anoikis, as a programmed cell death, is activated during tumorigenesis to remove detected cells from ECM (Adeshakin et al., 2021). As early as 2000, anoikis-mediated apoptosis has been reported to mediate gastric epithelial homeostasis by affecting the renewal of gastric epithelium during physiological conditions (von Herbay and Rudi, 2000). Gastric cancer cells that have acquired the ability to resist anoikis, can be survival and metastasis through the abdominal cavity or lymphatic/circulatory system far away without ECM contact (Simpson et al., 2008). In 2014, whole genome sequencing and comprehensive molecular profiling of gastric cancer showed that anoikis resistance was one of the driving factors in gastric cancer (Wang et al., 2014a). Several anoikis resistance related signal pathways have been reported in GC, including RhoA pathway, Wnt/β-catenin signaling pathway and EGFR signaling pathway, and these pathways promote angiogenesis, lymph node metastasis and peritoneal metastasis of gastric cancer cells (Wang et al., 2014b; Du et al., 2021; Li et al., 2022).

Long-stranded non-coding RNA (lncRNA) is defined as a non-coding RNA molecule whose length exceeds 200 nucleotides (Schmitt and Chang, 2016). With the development of sequencing technology, it has shown that lncRNAs are involved in various biological progresses including transcriptional regulation, RNA editing, and post-transcriptional regulation of many genes (Sun et al., 2018). In recent studies, lncRNAs have been presented being closely relevant to cell proliferation, migration, invasion and apoptosis, and are also involved in carcinogenesis, as a key regulatory factor for tumor occurrence, including GC (Huarte, 2015; Shih and Kung, 2017; Tang et al., 2017). In GC, the lncRNAs play an essential role in many processes, includes predicting the recurrence, metastasis, chemotherapy resistance and overall survival rate (Dastmalchi et al., 2022; Gong et al., 2022). LncRNA has been also used as an oncogene or tumor suppressor in gastric cancer (Zheng et al., 2022). However, the effect of anoikis-related lncRNA has yet to be reported in GC.

**FIGURE 1:**
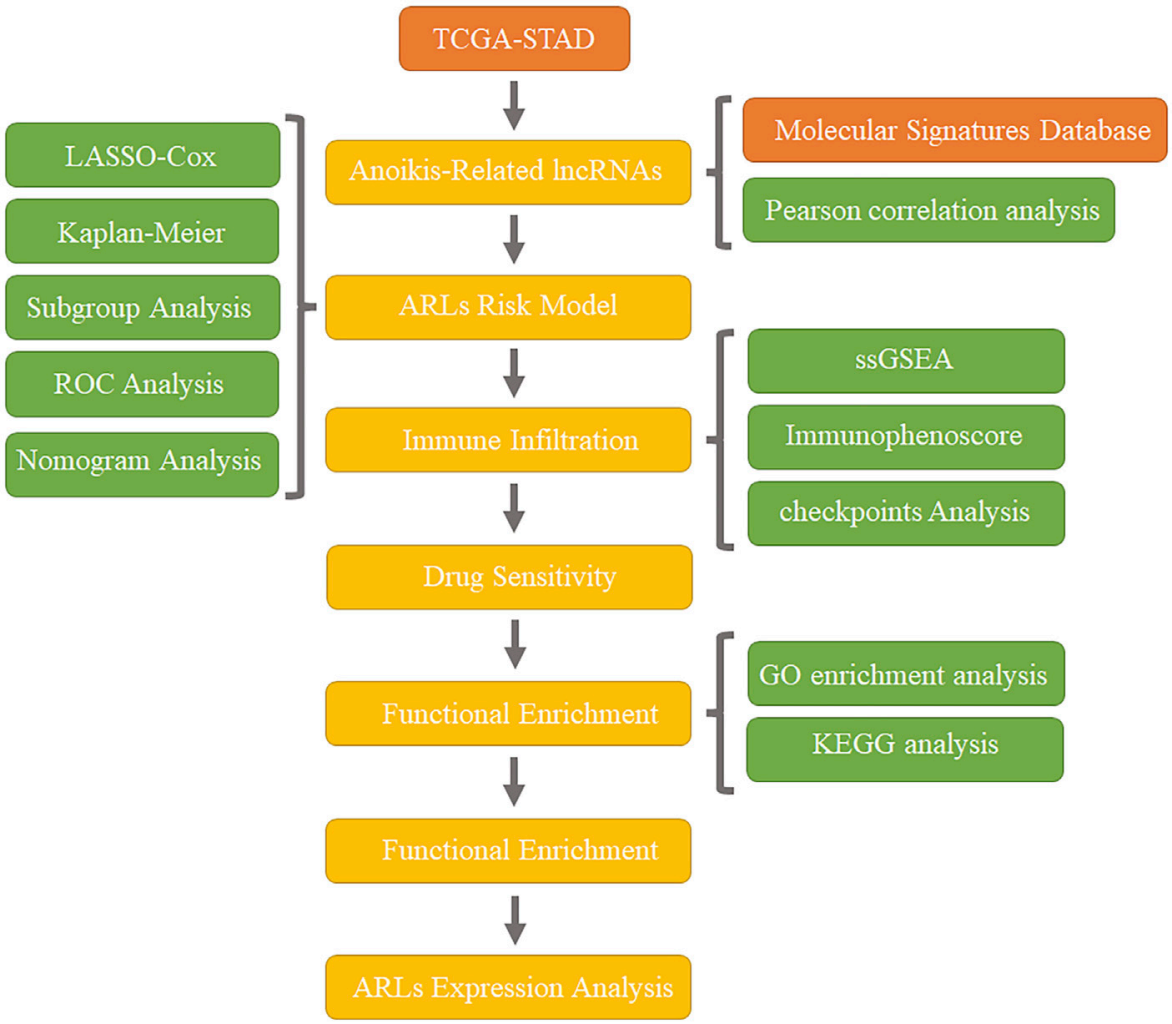
Workflow.

In this research, we established a STAD prognostic model based on five anoikis associated lncRNAs (ARLs) according to the TCGA database, and tested the accuracy of the model in prognosis prediction. Subsequently, we comprehensively discussed the mechanism of action and clinical significance of this prognostic model through clinical subgroup analysis, tumor mutation burden (TMB) detection, drug sensitivity analysis, immune infiltration, pathway enrichment analysis and other methods. The establishment of this model provides not only a new assessment method for the risk of distant metastasis of STAD, but also a new perspective on the individualized and precise therapy of STAD. Workflow is shown in Figure 1.

## Materials and methods

### Data collection

Open gene expression matrix with complete clinical characteristic information of 350 STAD patients was obtained from The Cancer Genome Atlas database (TCGA) (https://portal.gdc.cancer.gov/) by Perl scripts. Then, the expressions of mRNA and lncRNA were annotated and classified by utilizing the ensembles human genome browser GRCh38.p13 (http://asia.ensembl.org/index.html). All data involved in this study were obtained from the public database, and gained approval from the Ethics Committee. The written informed consents from patients were not required.

### Identification of anoikis-related long non-coding RNAs

A total amount of 34 anoikis-related genes (ARGs) was obtained from the Molecular Signatures Database (https://www.gsea-msigdb.org/gsea/). Next, the expressions of ARGs were extracted by Perl scripts, and then Pearson correlation analysis was performed to identify the anoikis-related lncRNAs. With a threshold setting at |correlation coefficient| > 0.4 and *p*-value <0.001, 298 anoikis-related lncRNAs (ARLs) were identified as STAD.

### Construction of ARLs risk model

According to the results of univariate Cox regression, the least absolute shrinkage and selection operator (LASSO) algorithm was applied to identify the ARLs associated with overall survival (OS) rate by R package “glmnet.” Then, multivariate Cox regression was used to identify the prognostic ARLs and the risk model was constructed. The risk score of each sample was calculated out by the following formula: = (−0.87 × TSPOAP1-AS1 expression) + (2.62 × CCNT2-AS1 expression) + (0.64 × LINC01094 expression) + (−1.24 × AL033527.2 expression) + (0.43 × LINC00460 expression). The STAD samples were divided into low- and high-risk groups in accordance with the median value of risk scores, and Kaplan-Meier survival curve was conducted to estimate the difference of overall survival rates between two groups.

### Validation of ARLs risk model in TCGA database

350 STAD samples from TCGA database were classified into the training and test cohorts in the proportion 7:3, that is, 245 samples in the training cohort and 105 samples in the test cohort. The risk score of each sample was, respectively calculated in both cohorts. According to the median of risk scores, all samples were divided into low- and high-risk groups.

### Independence evaluation of ARLs risk model

In order to investigate the index independence of ARLs risk model on STAD, univariate and multivariate Cox regression analyses were performed. A nomogram model was constructed according to ARLs risk model and clinicopathological characteristic with “rms” R package. The diagnostic accuracy of the ARLs risk score was evaluated by using the R package “pROC.” The prognostic capability of the risk model was evaluated by time-dependent receiver operating characteristic (ROC) curve.

### Evaluation of tumor microenvironment cells in patients with STAD

ESTIMATE score, stromal score, and tumor purity were calculated *via* “estimate” R package. A single sample gene set enrichment analysis (ssGSEA) algorithm was performed to assess the infiltration level of 23-types of immune cells *via* the “GSVA” R packages.

### Immunophenoscore and immune-checkpoint analysis

Immunophenoscore (IPS) of each STAD patient was obtained from the TCIA database (https://tcia.at/home). The expressions of immune checkpoints including CD200, NIRP1, PDCD1LG2, PD-L1, and CD86 were extracted from the TCGA using Perl scripts.

### Functional enrichment and drug sensitivity analysis

The “limma” R package was used to identify the differential expression genes (DEGs), and the *p*-value was adjusted by “FDR” method (|Fold Change| ≥ 2 and FDR <0.05). Kyoto Encyclopedia of Genes and Genomes (KEGG) and biology process (BP) analysis were performed to enrich DEGs into pathways using the “clusterProfiler” R package. Based on the Genomics of Drug Sensitivity in Cancer (GDSC) database, the IC50 of antineoplastic drugs in low- or high-risk group was investigated *via* R package “pRRophetic.”

### Statistical analysis

R software (version 4.1.2) was used to perform all statistical analyses. Wilcoxon rank-sum test was used to analyze differential functions between the two groups, and was statistically significant (*p* < 0.05).

### qRT-PCR

Total mRNA from tissues was extracted by Trizol/phenol/chloroform method and treated with DNase to eliminate genomic DNA. Reverse transcription was performed according to the HiScript III All-in-One RT SuperMix (R330-01, Vazyme, China) one-step kit instructions. qPCR experiments were carried out using ChamQ universal SYBR qPCR Master Mix (Q711-02, Vazyme, China), and ACTIN was used as the internal parameters. LightCycler96 was (Roche, Germany) used to get the curve. The procedures were as follows: 1) 5min at 95°C; 2) 40 cycles of 30 s at 95°C and 10 s at 60°C.

TSPOAP1-AS1

forward primer: 5′- CCT​GGA​GAC​TTC​ACG​CCA​AT -3′

reverse primer: 5′- GAG​CCC​ATC​TGA​ACA​CAG​C -3′

CCNT2-AS1

forward primer: 5′- TTA​CGG​ATG​AGG​GAC​CAC​GG -3′

reverse primer: 5′- CAG​AGA​AAA​GCA​GTT​TCC​CCA -3′

LINC01094

forward primer: 5′- TCC​CTT​CCA​CAG​AGA​AGG​CT-3′

reverse primer: 5′- AGG​TTG​ACA​CAT​CTC​GCC​TG -3′

AL033527.2

forward primer: 5′- CCA​GTA​CTG​ATC​CAG​CTT​CG-3′

reverse primer: 5′- GGG​CTG​AGT​TAG​TAG​AGG​CAT -3′

LINC00460

forward primer: 5′- GGC​ATT​GTA​GAA​AGA​CTG​AGC​G-3′

reverse primer: 5′- TAG​CAT​ACG​AAT​TTG​GGT​GGG-3′

ACTIN

forward primer: 5′- CAC​CAT​TGG​CAA​TGA​GCG​GTT​C-3′

reverse primer: 5′- AGG​TCT​TTG​CGG​ATG​TCC​ACG​T-3′

## Results

### Construction of the anoikis-related lncRNAs (ARLs) signature

**FIGURE 2:**
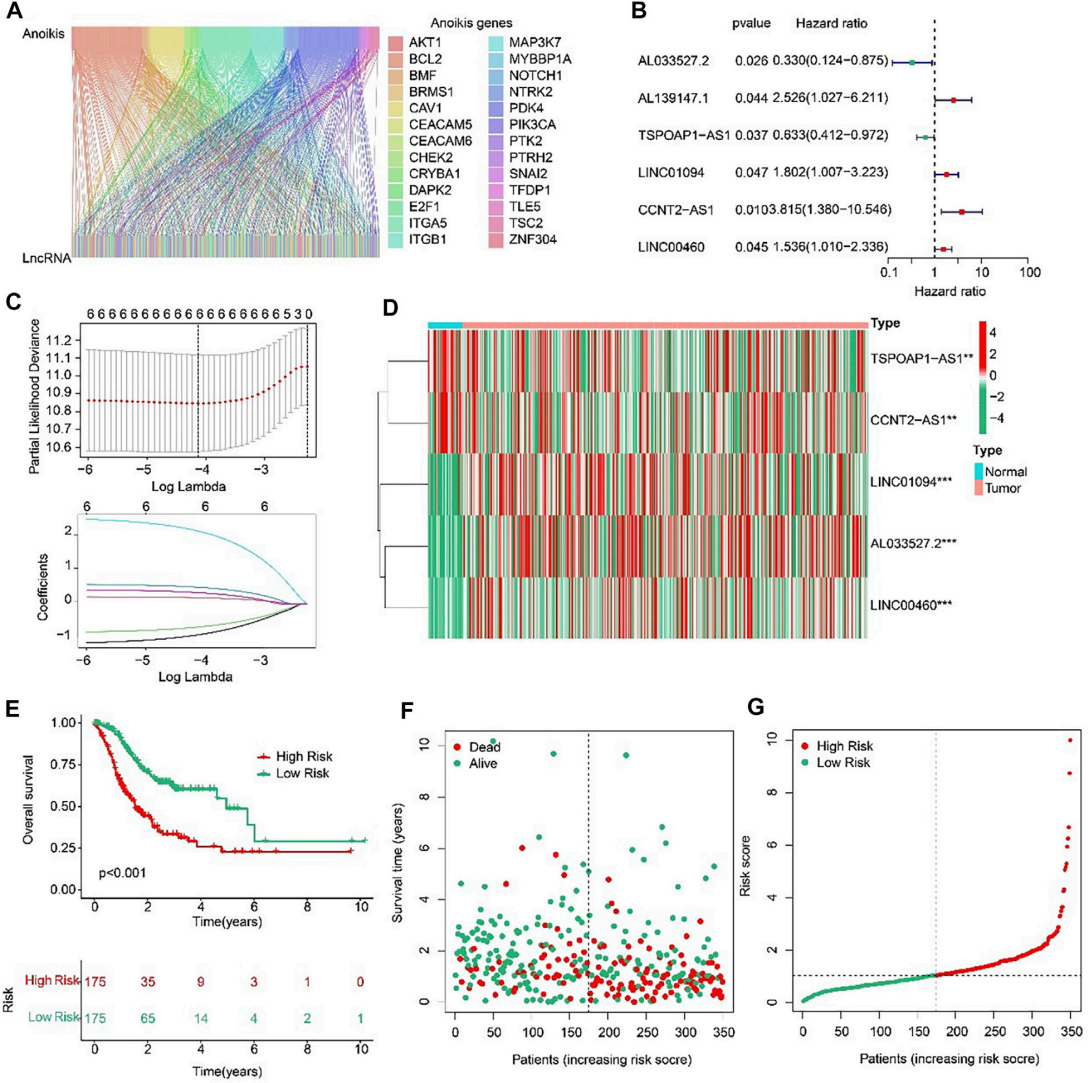
Risk model construction based on the prognostic ARLs in STAD. **(A)** The Sankey diagram presents the detail connection between ARGs and ARLs. **(B)** Univariate Cox regression analysis of ARLs. **(C)** LASSO regression analysis shows the minimum lambda and optimal coefficients of prognostic ARLs. **(D)** Heatmap of five prognostic ARLs in normal and tumor groups. **(E)** The Kaplan-Meier survival curve of the OS rate in STAD patients with different risk score. **(F, G)** The classification of the STAD patients according to the ARLs risk model and the scatter dot plot shows the correlation of the ARLs prognostic signature and survival time.

Sankey diagram indicated the relationship between anoikis-related genes (ARGs) and anoikis-related lncRNAs (ARLs) (Figure 2A). Based on the univariate Cox regression analyses, six prognostic ARLs associated with OS rate for STAD were identified *via* the least absolute shrinkage and selection operator (LASSO) analysis (Figures 2B, C). According to the multivariate Cox regression analyses, five prognostic ARLs differently expressed in tumor and normal groups, which could independently evaluate the prognosis of STAD and also were selected to construct the risk model (Figure 2D). The STAD patients were ranked and divided into the low- and high-risk groups according to the median of five ARLs prognostic signature. The Kaplan-Meier survival analysis showed that the patients with high-risk scores had a lower OS rate by comparison with low-risk score group (Figures 2E–G).

### Validation of the ARLs prognostic signature

**FIGURE 3:**
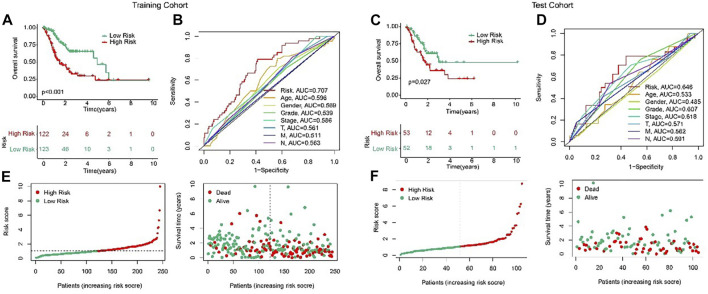
Validation of ARLs prognostic signature in STAD. Risk model construction based on the ARLs prognostic signature in training cohort **(E)** and test cohort **(F)**. The Kaplan-Meier survival curve analysis of the STAD patients with low- and high-risk score in the training cohort **(A)** and test cohort **(C)**. ROC curve shows the AUC of ARLs prognostic signature and other clinicopathological characteristics in the training cohort **(B)** and test cohort **(D)**.

In order to evaluate the accuracy of the anoikis-related lncRNAs (ARLs) prognostic signature in the prognosis prediction for the STAD patients, the patients were randomly classified into the training cohort and test cohort in the proportion 7:3. Then, according to the median value of risk scores, the patients were classified into the low- and high-risk groups in the both cohorts. The AUC of risk model shown in Figures 3B, D were 0.707 and 0.646, respectively in training and test cohorts. The scatter dot plot indicated that the risk score was inversely associated with survival time (Figures 3E, F). Kaplan-Meier survival results indicated that the patients with high-risk scores had a lower OS rate by comparison with those with low-risk scores in both cohorts (Figures 3A, C). These results demonstrated that the risk model could accurately evaluate the prognosis of STAD patients.

**FIGURE 4:**
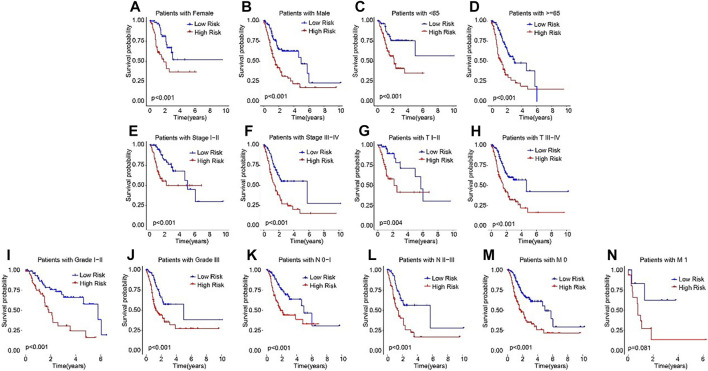
The Kaplan-Meier survival analysis of STAD patients in the different clinicopathological characteristics subgroups. The Kaplan-Meier survival curve analysis shows the OS rate of patients in the low- and high-risk groups among the **(A, B)** Gender (female vs. male); **(C, D)** Age (age >65 vs. age ≤65); **(E, F)** Stage (stage 0–1 vs. stage 2–4); (Gil et al., 2104); **(G,H)** T (T 0–1 vs. T 2–4); **(I, J)** Grade (G 1–2 vs. G 3); **(K, L)** N (N 0–1 vs. N 2–3); and **(M, N)** M (M 0 vs. M 1).

Subsequently, subgroup analysis was performed to explore the prognostic value of the ARLs risk model. On the basis of the calculated risk scores, the STAD patients were classified into the low- and high-risk groups among the different clinicopathological characteristics. The Kaplan-Meier survival analysis showed that the OS rate of patients in the low-risk group was significantly higher than high-risk group in female, male, age <65, age ≥65, stage I-II, stage III-IV, T I-II, T III-IV, grade I-II, grade III, N0-1, N2-3, M0, and M1 (Figures 4A–N). These results demonstrated that the ARLs-based risk model could accurately evaluate the survival probability of STAD patients.

### Anoikis-related lncRNAs (ARLs) risk model was an independent prognosis indicator

**FIGURE 5:**
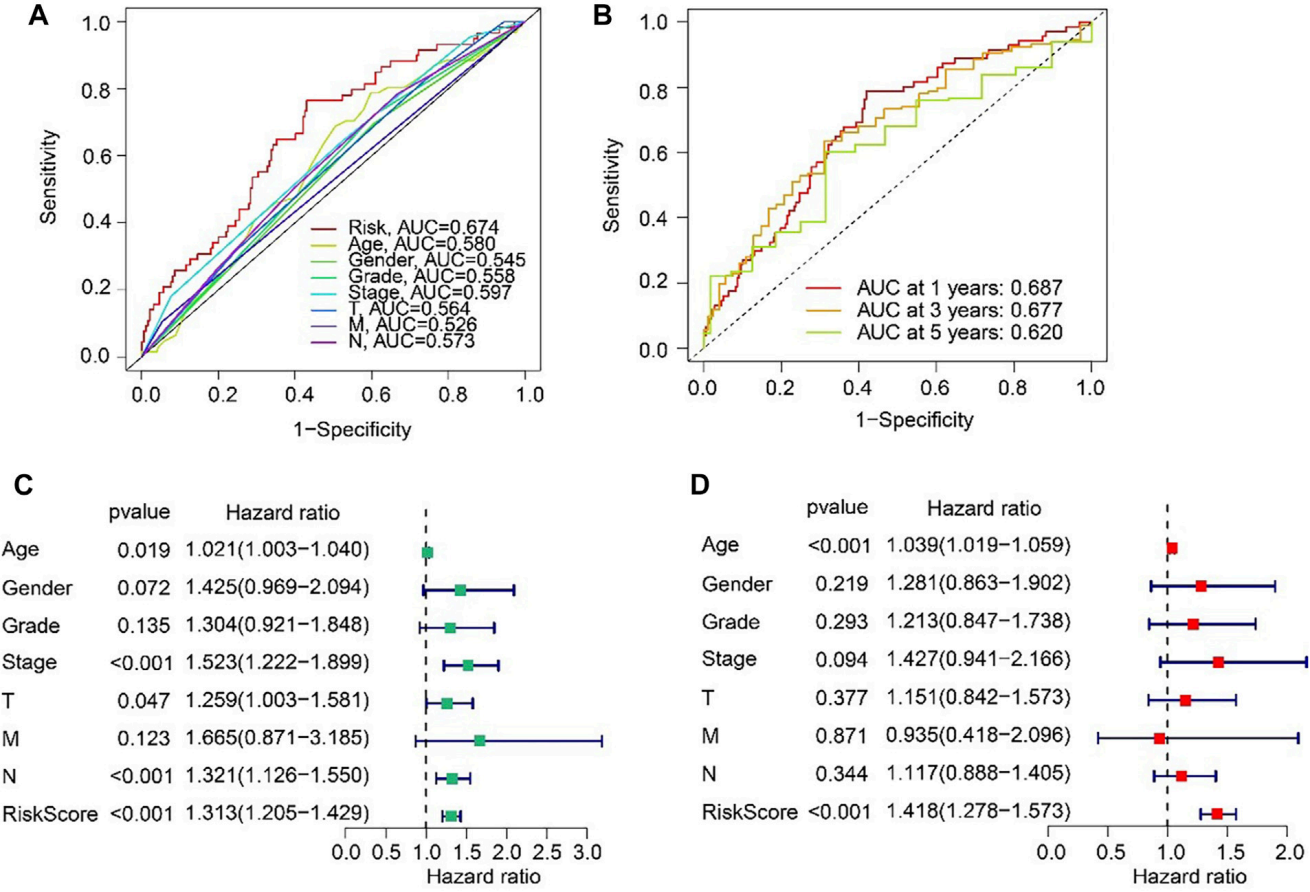
Independent prognosis analysis based on the ARLs prognostic signature and clinicopathological characteristics. **(A)** ROC curve shows the AUC of ARLs prognostic signature and other clinicopathological characteristics. **(B)** Time-dependent ROC curve shows the AUC at 1-, 3-, and 5-years. **(C)** Univariate Cox regression analysis. **(D)** Multivariate Cox regression analysis.

The ROC curve indicated that the AUC of risk score was 0.674, suggesting a satisfactory stability of the ARLs prognostic signature (Figure 5A). Besides, the time-dependent ROC curves indicated that the AUC of 1-, 3-, and 5-year was 0.687, 0.677, and 0.620, respectively (Figure 5B). Next, univariate and multivariate Cox regression analyses were utilized to evaluate the risk score based on the ARLs, that was as an independent prognosis predictor for STAD. Univariate Cox regression analysis showed that stage (HR = 1.523, *p* < 0.001), T (HR = 1.259, *p* = 0.047), N (HR = 1.321, *p* < 0.001), and risk score (HR = 1.313, *p* < 0.001) were closely correlated with OS rate of STAD (Figure 5C). Multivariate Cox regression analysis indicated that risk score (HR = 1.418, *p* < 0.001) was an independent prognosis indicator for STAD (Figure 5D).

### Nomogram of ARLs prognostic signature and clinicopathological characteristics

**FIGURE 6:**
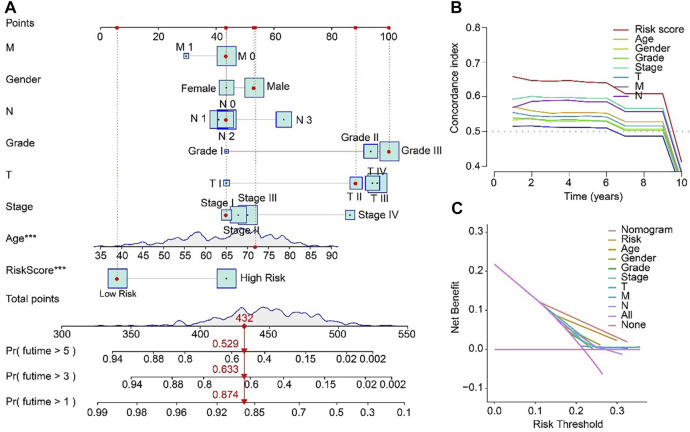
Nomogram model based on risk score for STAD patients in TCGA. **(A)** Nomogram construction of the ARLs prognostic signature and clinicopathological characteristics. **(B)** Concordance index and **(C)** Decision curve analysis (DCA) for the nomogram.

For the purpose of predicting the 1-, 3-, and 5-year survival probability of STAD patients, a nomogram based on the ARLs prognostic signature and clinicopathological characteristics was established (Figure 6A). The concordance index (C-index) of the nomogram was higher than other clinical features (Figure 6B). Compared with risk, age, gender, grade, stage, T, M and N, the DCA results of nomogram had advanced net benefits, which demonstrated that nomogram model had superior clinical prognostic worth than other indicators (Figure 6C). These results were a further proof of the accuracy and reliability of the nomogram based on the ARLs prognostic signature to evaluate the survival of STAD patients.

### Relationship between the ARLs risk model and immune cell infiltration

**FIGURE 7:**
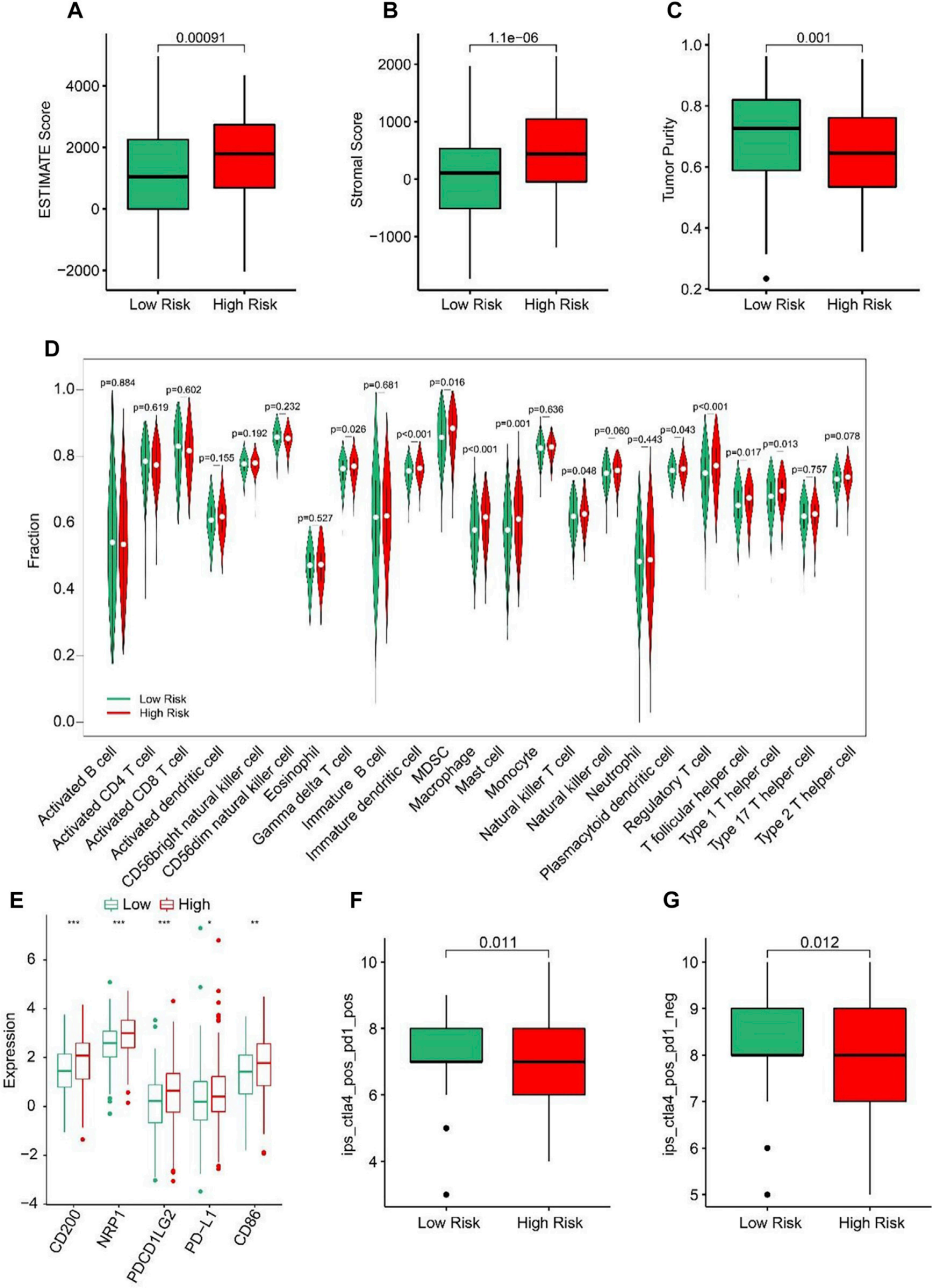
Immune infiltration landscape of patients in the low- and high-risk group. **(A)** ESTIMATE score. **(B)** Stromal score. **(C)** Tumor purity. **(D)** The proportion of 23-type cells of patients in low- and high-risk groups. **(E)** The expression of immune checkpoint inhibitor in the low- and high-risk groups. **(F, G)** Immunophenoscore (IPS) *p < 0.05, **p < 0.01, ***p < 0.001.

ESTIMATE algorithm results indicated that the STAD patients in high-risk group had higher ESTIMATE and stromal scores but lower tumor purity than those in low-risk group (Figures 7A–C). The results of ssGSEA suggested that the fractions of Gamma delta T cells, Immature dendritic cells, MDSC, Macrophage, Mast cells, NK T cells, Regulatory T cells, T follicular helper cells, and Type1 T helper cells were higher in high-risk group (Figure 7D). These findings demonstrated that the ARLs risk model is closely associated with the immune infiltration of STAD. Besides, the expression of immune checkpoints showed that CD200, NRP1, PDCD1LG2, PD-L1, and CD86 were all upregulated in high-risk group (Figure 7E). IPS results suggested that the patients with low-risk score showed a promising response to anti-CTLA-4, anti-PD-1 and anti CTLA-4/anti-PD-1, illustrating a better benefit for immunotherapy of STAD patients in the low-risk group (Figures 7F, G). Taken together, these results provided an innovation insight for the future individualized precise therapy for STAD patients in different risk subgroups.

### Correlation analysis of risk score and drug sensitivity

**FIGURE 8:**
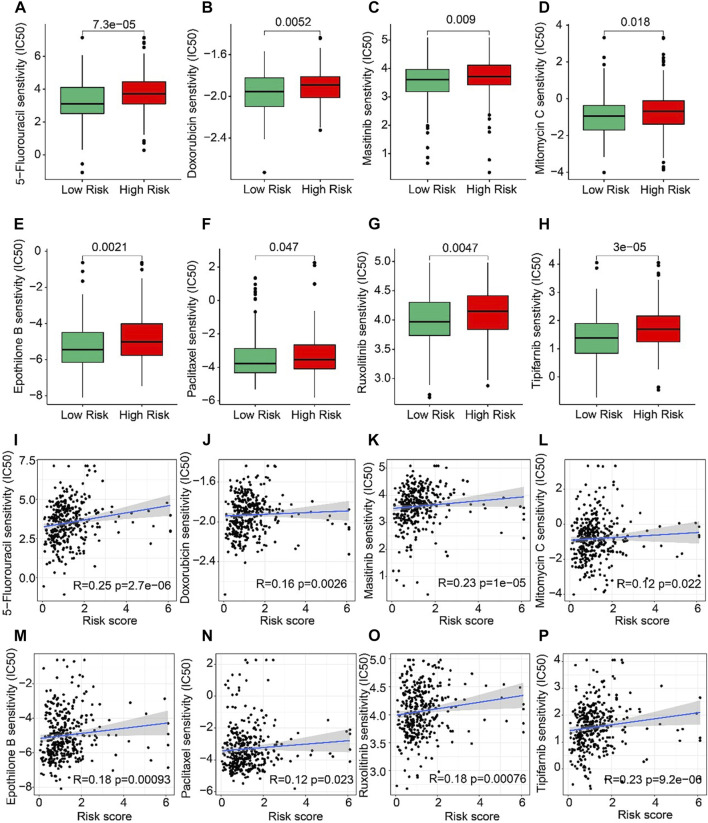
Drug sensitivity of patients in the low- and high-risk group. **(A)** 5-Fluorouracil, **(B)** Doxorubicin, **(C)** Masitinib, **(D)** Mitomycin C, **(E)** Epothilone, **(F)** Paclitaxel, **(G)** Ruxolitinib, and **(H)** Tipifarnib. Zhang et al. (2022) **(I–P)** Correlation analysis of the risk score and drug sensitivity (IC50).

The correlation between the risk score and drug sensitivity was analyzed. As shown in Figures 8A–H, compared with the low-risk group, the high-risk group had higher IC50 of 5-Fluorouracil, Doxorubicin, Masitinib, Mitomycin C, Epothilone, Paclitaxel, Ruxolitinib, and Tipifarnib. The risk score was positively correlated with 5-Fluorouracil (R = 0.25, *p* = 2.7e-06), Doxorubicin (R = 0.16, *p* = 0.0026), Masitinib (R = 0.23, P < 1e-15), Mitomycin C (R = 0.12, *p* = 0.022), Epothilone (R = 0.18, *p* = 0.00093), Paclitaxel (R = 0.12, *p* = 0.023), Ruxolitinib (R = 0.18, *p* = 0.00076), Tipifarnib (R = 0.23, *p* = 9.2e-06) (Figures 8I–P). Above results indicated that the sensitivity of different drugs for STAD in two risk subgroups varied wildly, suggesting the importance of individualized therapy in the future.

### Functional enrichment and tumor mutation burden analysis

**FIGURE 9:**
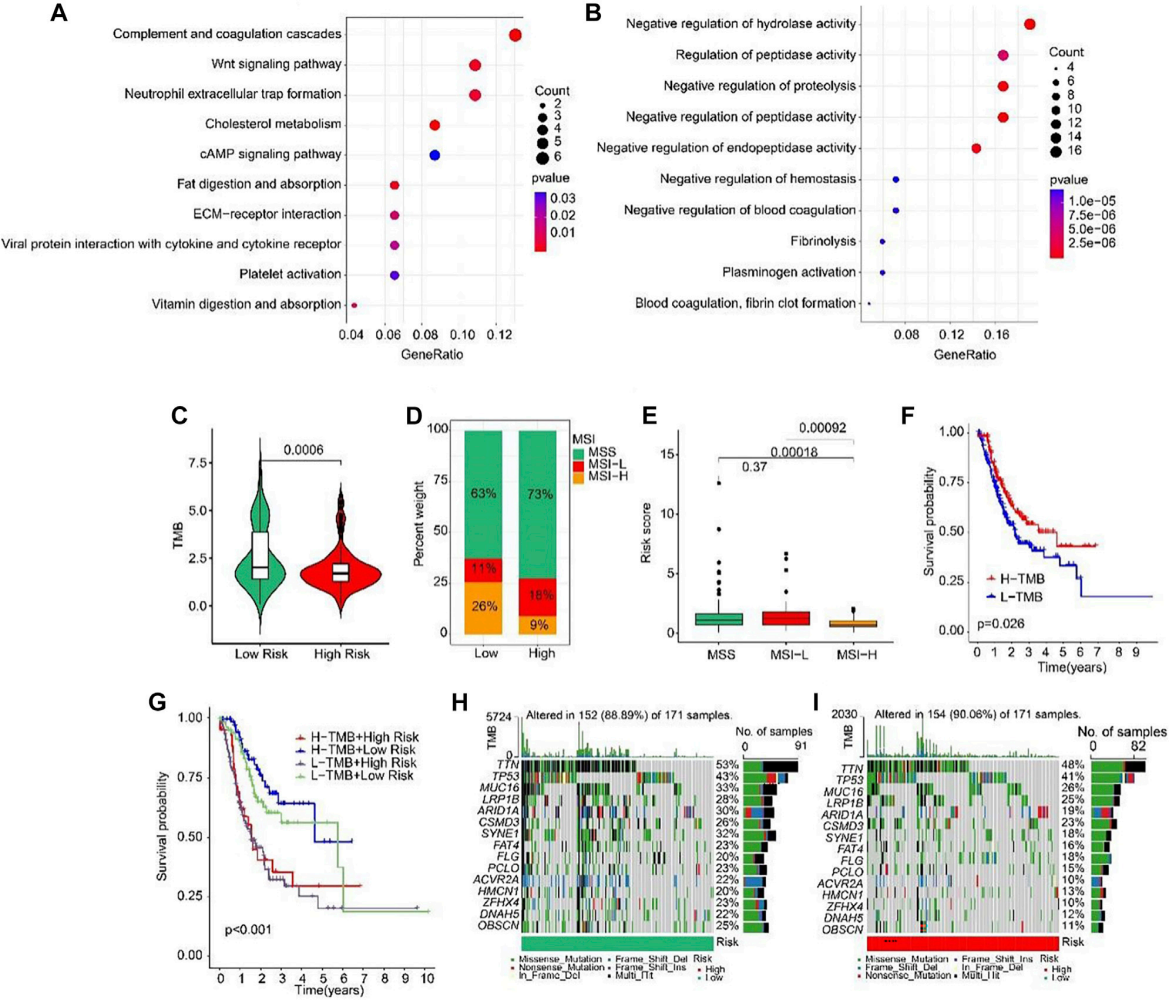
Tumor mutation burden and Functional enrichment analysis of DEGs in low- and high-risk group. **(A)** GO analysis of DEGs in low- and high-risk groups. **(B)** KEGG analysis of DEGs in low- and high-risk groups. **(C)** Tumor mutation burden between low- and high-risk groups. **(D)** MSI percent between low- and high-risk groups. **(E)** Risk score between low- and high-risk groups. **(F)** The Kaplan-Meier survival curve shows the OS rate of patients in H-TMB and L-TMB groups. **(G)** The Kaplan-Meier survival curve shows the OS rate of patients in H-TMB + high-risk, H-TMB + low-risk, L-TMB + high risk, and L-TMB + low risk groups. **(H, I)** The waterfall plot of somatic mutation landscape of low- and high-risk groups.

Biological process of GO enrichment analysis revealed that the DEGs between high-risk and low-risk groups were enriched in complement coagulation cascades, Wnt signaling pathway, and neutrophil extracellular trap formation (Figure 9A). KEGG analysis showed that the DEGs were negative regulation of hydrolase activity and regulation of peptidase activity (Figure 9B). Tumor mutation burden (TMB) were analyzed in both high- and low-risk groups. The TMB in high-risk group was lower than that in low-risk group (Figure 9C). Further, the percent of MSS and MSI-L were higher in high-risk group than that in low-risk group (Figure 9D). Besides, the risk scores of MSS and MSI-L were higher than MSI-H (Figure 9E). The survival probability in high tumor mutation burden (H-TMB) is higher than low tumor mutation burden (L-TMB) (Figure 9F). Interestingly, patients were divided into four groups according to the risk score and TMB, and we found that the survival probability of H-TMB + low risk is highest (Figure 9G). In addition, the top 15 genes with the highest mutation frequency were visualized in high-risk and low-risk groups (Figures 9H, I).

### Validation of the expression levels of lncRNAs of prognostic signature

**FIGURE 10:**
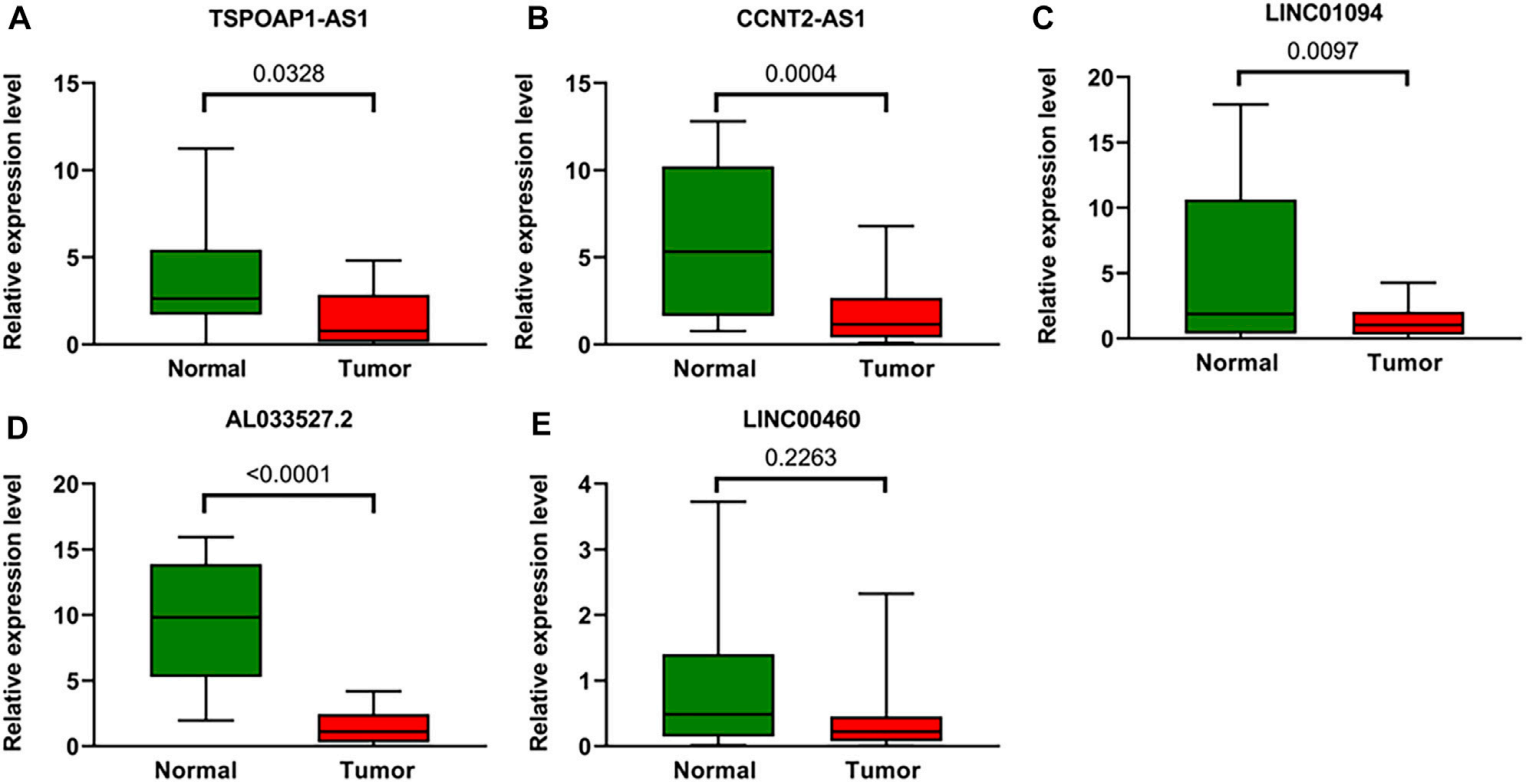
Validation of the expression Levels of lncRNAs of prognostic signature. **(A–E)** TSPOAP1-AS1, CCNT2-AS1, LINC01094, AL033527.2, and LINC00460 expressions in gastric cancer tissues compared with corresponding normal tissues from patient samples.

To further verify the accuracy of ARLs, the expression levels of lncRNA TSPOAP1-AS1, CCNT2-AS1, LINC01094, AL033527.2 and LINC00460 were detected in 20 gastric cancer tissues and 20 adjacent normal tissues by qRT-PCR. Compared with normal tissues, four ARLs were downregulated in gastric cancer tissues (Figures 10A–D). LINC00460, though not statistically significant, showed the same trend (Figure 10E).

## Discussion

At present, although there is no risk stratification model associated with anoikis in STAD, resistance to anoikis is a landmark event of cancer cell migration, and resistance to anoikis is crucial for the systemic spread of cancer cells (Du et al., 2021; Zeng et al., 2022). Since the prognosis of advanced STAD with distant metastasis is significantly worse than that of early STAD (Harada et al., 2017), the study on anoikis is of great significance for the prognosis of GC. This study successfully established a prognostic model of STAD based on five ARLs, further demonstrating the clinical application potential of ARLs in STAD. Among these five ARLs, only AL033527.2 and LINC00460 have been reported as prognostic biomarkers in GC (Tang et al., 2021), TSPOAP1-AS1 has been identified as a prognostic factor in pancreatic cancer (Wang et al., 2022) which may be involved in IFN signaling to influence viral replication (Li et al., 2021a), and CCNT2-AS1 is regarded as a prognostic factor for high-grade renal cell carcinoma (Liu et al., 2019) while LINC01094 has not been reported in any cancer studies. In general, as a new prognostic risk model, the studies on these five ARLs are superficial and further research is necessary in the future.

Our immune infiltration results showed that patients at high risk of ARL had higher levels of regulatory T cell (Treg) infiltration. In the tumor microenvironment, Tregs are involved in homeostasis regulation and tumor immune escape. An increase in Treg cells in the tumor immune microenvironment demonstrates a poor prognosis (Pires et al., 2020). CD4^+^ T cells, CD8^+^ T cells, antigen presenting cells (APC), monocytes and macrophages were inhibited by Treg enrichment. Tumor cells were thus able to multiply more rapidly under the immunosuppressive condition of Tregs enrichment (Pires et al., 2020). Although no correlation between anoikis and Treg has been reported, ECM immune remodeling has been observed in patients with unresponsive tumors with poor Treg-targeting effect (Bollyky et al., 2011). The role of hyaluronic acid (HA), an important component of ECM, in supporting Treg homeostasis has been reported. HA promotes Treg function and expression of Treg marker proteins Foxp3 and IL-10 through p38 and ERK 1/2 signaling (Bagheri et al., 2017). A correlation between Treg and HP infection has been reported in GC. The expression level of FOXP3^+^ Tregs in HP infected patients was significantly higher than that in non-infected patients (Chen et al., 2010). The eradication of HP led to the downregulation of Tregs mRNA (Gil et al., 2014). In patients with *Helicobacter pylori* (HP) gastritis, the reduction of Th17/Treg ratios can enhance bacterial persistence and lead to chronic active gastritis (Wu et al., 2011). It has also been reported that increased infiltration of Tregs in gastric mucosa can suppress the immune response and lead to the persistence of HP infection (Lundgren et al., 2005; Zhang et al., 2022). Tregs infiltration has also been shown to be associated with poorer outcomes in GC patients (Perrone et al., 2008; Shen et al., 2010; Kim et al., 2011). Therefore, Treg is an important link in the occurrence and development of GC. GC immunotherapy targeting Tregs is also currently in clinical trials. However, no significant improvement in GC clinical effect has been seen by targeting Treg (Pires et al., 2020). The efficacy of Treg-related treatments needs to be further explored.

In cancer cells, anoikis resistance is regulated by different genes and signaling pathways (Cardone et al., 1997; Zhang et al., 2021). Our pathway enrichment analysis showed that Wnt signaling pathways were involved in anoikis-related GC risk stratification. The Wnt signaling pathway is highly involved in cancer development and is essential for a variety of cellular functions including invasion and metastasis (Xue et al., 2022). Dysregulation of the Wnt pathway is observed in approximately 50% of in GC tissues (Ooi et al., 2009). The activation of multiple Wnt pathway members in GC was significantly increased and positively correlated with tumor stage, depth of tumor invasion and degree of lymph node metastasis (Koushyar et al., 2020). Wnt pathway has an important effect on GC distant metastasis. Activated Wnt/β-catenin signaling induces epithelial-mesenchymal transition and promotes lymph node metastasis in GC patients (Li et al., 2022). The experimental results showed that compared with the control GC cell line, the injection of Wnt5a knockdown metastatic GC cells into the spleen of nude mice significantly reduced the number of liver metastatic nodules (Yamamoto et al., 2009). Antibody targeted inhibition of Wnt5a against the Wnt ligand significantly reduced the number of metastatic liver lesions (Hanaki et al., 2012). Therefore, Wnt inhibitors may be effective anti-metastases for GC. There are several ongoing clinical trials targeting the Wnt pathway for advanced distant metastatic GC patients. Like the Wnt pathway, anoikis resistance is also a signature event of cancer cell migration (Simpson et al., 2008). Several evidences hint at the correlation between anoikis resistance and Wnt pathway. A Wnt signaling receptor, FZD7, has been shown to be critical for anoikis resistance in ovarian cancer (Tan et al., 2019). In gastric stem cells, Wnt5a activated RhoA to reduce anoikis resistance (Hayakawa et al., 2015). Based on these studies, the in-depth study of the internal mechanism of anoikis resistance and Wnt pathway is expected to provide new ideas for further revealing of the GC cells metastasis mechanism.

The emergence of multiple drug resistance (MDR) in GC cells is one of the major challenges leading to treatment failure in clinical practice. It has been found that multiple lncRNAs are abnormally expressed in GC and promote MDR by regulating different target genes and signal pathways, including PCAT-1, SNHG5, GHET1, HOTAIR (Li et al., 2021b). And many genes and pathways are involved in this process, like PI3K/AKT, Wnt, NF-kB, and ABCB1 (a key multidrug-resistant gene). Our these data show that ARLs risk stratification has a significant effect on the resistance of first-line chemotherapeutic agents such as 5-fluorouracil (5-FU), doxorucin (DOX), and paclitaxel (PTX) for GC. Even though ARLs involved in risk stratification have not been reported, other lncRNAs have been reported to be associated with resistance to these drugs. LncRNA FAM83H-AS1 mediates resistance to 5-FU in GC cells by regulating the Wnt/β-catenin pathway (Wang et al., 2020). LncRNA MACC-AS1 regulates the fatty acid oxidation level of GC cells by regulating mesenchymal stem cells (MSCs) to realize the purpose of enhancing 5-FU resistance (He et al., 2019). LncRNA MRUL leads to DOX resistance in two multidrug-resistant GC cells by positively influencing the expression of ABCB1 (Wang et al., 2014a). LncRNA UCA1 endows GC cells with DOX resistance by influencing the apoptotic pathway involved in PARP1 and Bcl-2 (Shang et al., 2016). LncRNA ZFAS1 can stimulate the Wnt/β-catenin pathway to enhance PTX resistance in GC (Xu et al., 2018). Elevated lncRNA HOTAIR in drug-resistant GC cells may enhance PTX resistance in GC cells by inhibiting miRNA-217 (Wang et al., 2018). Based on these evidences, further combination of lncRNA therapeutic interventions and traditional chemotherapy drugs may be a promising treatment strategy for GC.

In summary, we developed a first STAD prognostic model based on five ARLs. This successful model could be used as a tool for GC analysis, such as immune infiltration, chemotherapy resistance and cell pathways, and provide a new idea for clinical individualized therapy and a potential therapeutic target for further research.

## Data Availability

The original contributions presented in the study are included in the article/supplementary materials, further inquiries can be directed to the corresponding authors.
